# Alpha-chloralose poisoning in cats: clinical findings in 25 confirmed and 78 suspected cases

**DOI:** 10.1177/1098612X221107787

**Published:** 2022-06-27

**Authors:** Cecilia Tegner, Sandra Lundgren, Kristoffer Dreimanis, Annica Tevell Åberg, Ulrika Windahl

**Affiliations:** 1 University Animal Hospital, Swedish University of Agricultural Sciences, Uppsala, Sweden; 2 Swedish National Veterinary Institute (SVA), Uppsala, Sweden; 3 Department of Medicinal Chemistry, Analytical Pharmaceutical Chemistry, Uppsala University, Uppsala, Sweden

**Keywords:** Alpha-chloralose, poisoning, intoxication, toxicosis, rodenticide

## Abstract

**Objectives:**

The aim of this study was to describe the clinical picture in cats with alpha-chloralose (AC) intoxication and to confirm AC in serum from suspected cases of AC poisoning.

**Methods:**

Suspected cases of AC poisoning were identified in patient records from a small animal university hospital from January 2014 to February 2020. Clinical signs of intoxication described in respective records were compiled, the cats were graded into four intoxication severity scores and hospitalisation time and mortality were recorded. Surplus serum from select cases in late 2019 and early 2020 was analysed to detect AC with a quantitative ultra-high performance liquid chromatography tandem mass spectrometry analysis, and the AC concentration was compared with the respective cat’s intoxication severity score.

**Results:**

Serum from 25 cats was available for analysis and AC poisoning was confirmed in all. Additionally, 78 cats with a clinical suspicion of AC intoxication were identified in the patient records, most of which presented from September to April. The most common signs of intoxication were ataxia, tremors, cranial nerve deficits and hyperaesthesia. The prevalence of clinical signs and intoxication severity differed from what has previously been reported, with our population presenting with less severe signs and no deaths due to intoxication. The majority had a hospitalisation time <48 h, irrespective of intoxication severity score.

**Conclusions and relevance:**

This study describes the clinical signs and prognosis in feline AC intoxication. There were no mortalities in confirmed cases, indicating that AC-poisoned cats have an excellent prognosis when treated in a timely manner. Recognition of AC intoxication as a differential diagnosis for acute onset of the described neurological signs in areas where AC exposure is possible may influence clinical decision-making and help avoid excessive diagnostic procedures. A severe clinical picture upon presentation could be misinterpreted as a grave prognosis and awareness about AC poisoning may avoid unnecessary euthanasia.

## Introduction

Alpha-chloralose (AC) is a compound with both excitatory and depressive effects on the central nervous system, in low and high doses, respectively. It is used as a rodenticide, avicide and as an anaesthetic for laboratory animals.^1–10^ AC-based rodenticides are approved in many countries and cause death by central nervous affection, leading to an inability to maintain homeostasis.^1,2^ For outdoor pets, accidental poisoning is a risk in areas where these products are used for pest control. Clinical signs of AC intoxication in several species have previously been reported,^1–11^ but publications on AC poisoning in companion animals are scarce.^12–15^ AC intoxication in the clinical setting is a presumptive diagnosis based on history, clinical signs and exclusion of other diseases with a similar presentation. Poisoning can be confirmed by demonstrating AC or its metabolites in blood or urine;^1,6,11–13,16^ however, the availability of commercial tests has so far been limited. As there is no antidote or otherwise specific treatment for AC intoxication, treatment is supportive and symptomatic, including monitoring and maintaining a normal body temperature, minimising external stimuli and, when indicated, anticonvulsants.^1,5,9^

Following the approval of use of AC rodenticides in Sweden in 2013, suspected cases of AC poisoning in outdoor cats have been anecdotally communicated within the veterinary community. In the autumn of 2019, there was a surge in suspected cases admitted to the University Animal Hospital, Small Animal Clinic (UDS) at the Swedish University of Agricultural Sciences in Uppsala and other small animal hospitals and clinics across the country. A collaboration between UDS and the Swedish National Veterinary Institute was initiated, leading to the development and validation of a novel quantitative ultra-high performance liquid chromatography tandem mass spectrometry (UHPLC–MS/MS) analysis for the detection of AC in feline blood.^16^

The objectives of this study were to describe clinical signs of feline AC poisoning, confirm intoxication through chemical analysis of blood samples from admitted cats and to investigate the association between the severity of intoxication and AC serum concentrations.

## Materials and methods

The UDS patient record database from January 2014 to February 2020 was reviewed to identify suspected cases of AC poisoning in client-owned cats presenting to the emergency clinic. The database did not include AC intoxication as a specific diagnosis, and the search was based on the following primary and/or secondary diagnoses: unspecified poisoning; rodenticide poisoning; signs of poisoning or intoxication; and signs of neurological disease. In most cases, the attending clinician had noted AC intoxication as the main differential diagnosis. Cats were included if the following criteria were fulfilled: (1) outdoor cat with possible exposure to AC according to history; (2) a clinical suspicion of AC intoxication; and (3) the presence of at least two signs of AC intoxication from a clinical case definition based on previous publications on AC poisoning.^1,2,9,12–15^ Signs included coma; stupor; somnolence; seizures; cranial nerve deficits, including vision impairment; ataxia; tremor; hyperaesthesia; hypothermia; bradycardia; bradypnoea or other respiratory alteration; hypotension and behavioural changes (see supplementary material for definitions of signs). Cats were excluded if there was a history of trauma, no possible risk of exposure (ie, indoor cats) or a previous diagnosis of neurological disease.

For each cat, clinical signs of intoxication were noted as either absent or present based on the patient record. Signs noted by the caregiver were also included, regardless of whether the sign was seen at presentation or during stationary care. A score of the overall clinical severity of intoxication was assigned to each cat and mortalities were noted. Ambulatory cats with few or mild signs were given scores of 1 or 2 and non-ambulatory cats with several and/or more severe signs were scored as 3 or 4 (Table 1). The identification of cases, documentation of signs and scoring of each case was performed by one clinician.

**Table 1:** Description of clinical signs with respect to intoxication severity score assigned to cats with suspected alpha-chloralose poisoning in 2014–2020 admitted to the University Animal Hospital, Small Animal Clinic, Uppsala, Sweden

Clinical signs	Score	Definition
		Ambulatory
Mild	1	Minor and/or fewer than four clinical signs
Moderate	2	Moderate and/or more than three clinical signs
		Non-ambulatory
Severe	3	Severe and more than five clinical signs except those mentioned for score 4
Very severe	4	Severe and more than five clinical signs, including coma, seizures and apnoea. Also including patients without coma, seizures or apnoea, but with severe affection of at least two of the following: systolic blood pressure (<80 mmHg), heart rate (<80 beats/min), respiratory rate (<10 breaths/min) or body temperature (<35°C)

Available surplus serum samples from 25 cats that had presented from October 2019 to February 2020 were analysed in order to detect AC. The cats were aged 5 months to 15 years (median 5 years). All but one (Maine Coon mixed-breed) were domestic shorthair or domestic longhair cats. Six cats were male castrated, seven male entire, nine female spayed and three female entire. There was no standardised treatment protocol, and treatment was dependent on the attending clinician. All patients in this group had been provided with supportive care with intravenous crystalloid fluids, warming when hypothermic and reduction of external stimuli (ie, dark and silent environment with minimal handling). Some patients had also been given a constant rate infusion (CRI) of intralipid (200 mg/ml; Fresenius Kabi) lipid emulsion to help bind circulating toxins, and patients with seizures had been treated with anticonvulsants as needed.

A novel validated UHPLC–MS/MS method with a lowest detection limit of 30 ng/ml was used.^16^ For each cat, the association between serum concentration of AC and intoxication severity score (1–4) was calculated using Spearman’s correlation. The duration of hospitalisation for each cat was noted and the median and range described for each severity score group. Outdoor cats admitted to the clinic with (n = 9) and without (n = 10) neurological signs but no clinical suspicion of AC poisoning were used as controls.

## Results

A total of 103 cases were extracted from patient records and 44 surplus serum samples from 25 cats with suspected AC poisoning were available for chemical analysis. AC was detected in all samples in a concentration range of 386–17,500 ng/ml and was not detected in any of the 19 controls. All samples had been stored at −20°C prior to analysis.

In the 25 confirmed cases, the most common clinical signs were ataxia and tremor, described in all cats (n = 25). Cranial nerve deficits (mainly vision impairment), hyperaesthesia, bradycardia, somnolence and behavioural changes were all described in ⩾60% of the cases (Table 2). Median duration of hospitalisation was 24 h (range 13–75) with no difference between severity scores. All but one cat (severity score 4) were hospitalised for <48 h, and all cats survived to discharge.

**Table 2:** Prevalence of clinical signs in 25 cats with confirmed alpha-chloralose poisoning admitted to the University Animal Hospital, Small Animal Clinic, Uppsala, Sweden in October 2019 and February 2020

Clinical signs	Number of cats (%)
Ataxia	25 (100)
Tremor	25 (100)
Cranial nerve deficits	24 (96)
Hyperesthesia	22 (88)
Bradycardia	20 (80)
Somnolence	20 (80)
Behavioural changes	15 (60)
Stupor	12 (48)
Hypothermia	10 (40)
Respiratory alteration	6 (24)
Hypotension	5 (20)
Coma	4 (16)
Seizures	2 (8)

**Figure 1:**
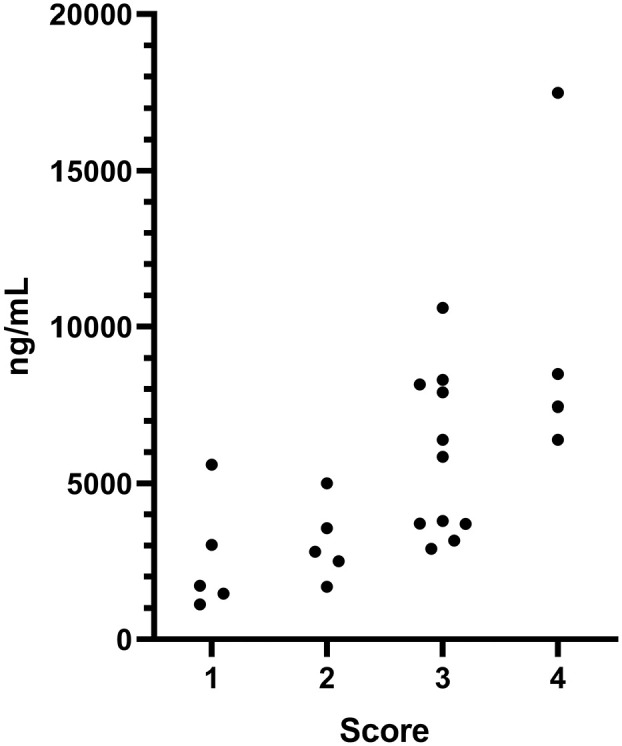
The highest detected concentration of alpha-chloralose (AC) in serum samples from 25 cats with AC concentration plotted against intoxication severity scores of 1–4 (*r* = 0.74; *P* <0.0001)

With regard to intoxication severity scores, five cats were scored as mild (1), five as moderate (2), 11 as severe (3) and four as very severe (4). The intoxication severity score correlated with the detected AC concentration (*r* = 0.74; *P* <0.0001 [Figure 1]). When several samples from the same individual were analysed, the sample with the highest AC concentration was used in the comparison.

Seventy-eight additional suspected cases were extracted from the UDS patient medical record database. With regard to intoxication severity, 15 were classified as mild (1), 24 as moderate (2), 22 as severe (3) and 17 as very severe (4). The prevalence of clinical signs was, overall, in agreement with the confirmed cases of AC poisoning. However, seizures were notably more common in this group, with 22% of suspected case records noting seizures vs 8% of confirmed cases (see Tables 2 and 3). There were no mortalities due to poisoning; however, nine of the cats in this group (11%) were euthanased at presentation, at the owners’ request.

**Figure 2:**
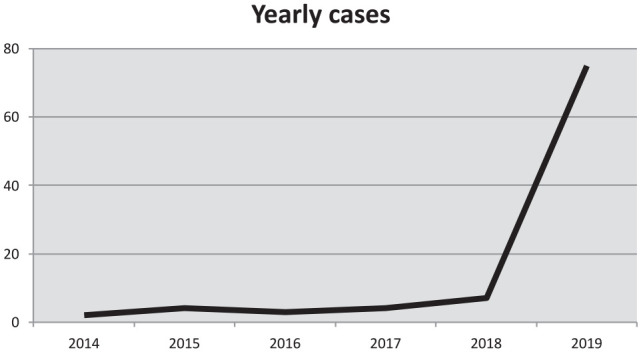
Number of cats presenting annually to University Animal Hospital, Small Animal Clinic, Uppsala, Sweden, with suspected alpha-chloralose poisoning in 2014–2019

Of all cases (n = 103), two presented in 2014, four in 2015, three in 2016, four in 2017, seven in 2018, 74 in 2019 (Figure 2) and nine in the first two months of 2020. In the years 2014–2019, 84/94 cats (89%) were admitted from September to April each year.

**Table 3:** Prevalence of clinical signs in 78 cats presenting with suspected alpha-chloralose poisoning admitted to University Animal Hospital, Small Animal Clinic, Uppsala, Sweden, between January 2014 and February 2020

Clinical signs	Number of cats (%)
Ataxia	76 (97)
Tremor	73 (94)
Cranial nerve deficits	67 (86)
Hyperaesthesia	61 (78)
Behavioural changes	52 (67)
Bradycardia	50 (64)
Somnolence	48 (62)
Hypothermia	33 (42)
Stupor	27 (35)
Seizures	17 (22)
Coma	8 (10)
Hypotension	8 (10)
Respiratory alteration	6 (8)

## Discussion

The present study aimed to contribute to the knowledge about AC intoxication in cats by describing the clinical presentation of a large group of cats with suspected AC poisoning admitted to a small animal university hospital.

AC poisoning was confirmed in 25 cats where blood samples were available for chemical analysis. The prevalence of clinical signs differed somewhat from what was previously reported by Segev et al,^12^ who reported a series of AC intoxication in both dogs and cats. The most common clinical signs observed by Segev et al in 13 cats were hypothermia, seizures, coma, hyperaesthesia, miosis and tremor.^12^ The clinical presentation was overall more severe in the report by Segev et al,^12^ with 46% of patients presenting comatose, and seizures noted in 54% of cats. This could reflect a difference in the definition of coma between studies. Clinically, severe hyperaesthesia may be very similar to partial seizures, with an overlap in the appearance and interpretation of the two, both between studies and between clinicians in the present study. Furthermore, while Segev et al reported an overall mortality of 15%, there were no mortalities in the cats with confirmed AC poisoning in the present study. Increasing public awareness about AC poisoning in cats in Sweden in 2019 may have influenced caregivers to seek veterinary care for cats with few or minor signs. This may have decreased the mortality and severity of signs of intoxication in this population as a result of receiving supportive care early in the progression of intoxication. However, the duration of hospitalisation in surviving cats was similar between our study and that which has been previously reported.^12^

Bradycardia was not reported by Segev et al,^12^ but was noted in 80% of our cats with confirmed poisoning. Tremor and ataxia, which were noted in all our cats with confirmed intoxication, was noted in 15% and 0%, respectively, in the study by Segev et al.^12^ At UDS, hypothermia has been considered a very common sign of AC poisoning. When normal endothermia is impaired, the ambient temperature will greatly influence body temperature. In mice, ambient temperatures below 15.6°C were shown to be associated with higher mortalities after AC bait ingestion.^1,2^ As most cats in the present study presented to the clinic in the colder months (September–April), hypothermia is potentially a lethal consequence of poisoning. However, only 40% of confirmed cats presented with hypothermia (ie, fewer than expected). In the report by Segev et al, hypothermia was the most common sign of intoxication, seen in 91% of the cats.^12^ It is possible that the normothermic cats in the present study were admitted prior to the development of hypothermia, and that it was prevented by the supportive care provided. Regardless, these findings illustrate the importance of not excluding AC poisoning based on normothermia.

Cats are more sensitive to AC than both dogs and humans, with a minimum lethal dose of 100 mg/kg vs 600–1000 mg/kg for dogs and >1000 mg/kg for humans.^2,8,9^ Hanriot and Richet (1893, cited in Balis and Monroe)^2^ reported the LD_50_ for cats and dogs to be 400–600 mg/kg orally, but injecting as little as 40 mg/kg could lead to convulsions. In an experimental model, a 5 mg/kg/h CRI maintained a stable level of surgical anaesthesia.^4^ To our knowledge, there have been no publications on the amount of intake, or AC serum concentration, where the first signs of intoxication can be expected in cats.

In the present study, it was not possible to determine with certainty how the cats had been exposed to AC. Several caregivers reported known baiting with AC either on their own property or in the vicinity. Many observed the onset of neurological signs shortly after the cat had eaten a rodent. Ingested rodent carcasses were occasionally detected radiographically in the stomach at the time of presentation and one of the confirmed poisoned cats vomited a mouse carcass at the clinic. Many bird species are also very sensitive to AC and secondary poisoning through ingestion of intoxicated birds cannot be excluded.^9^ Cats are also known dietary neophobes. A pilot study on the use of AC as a poison for feral cat control showed that the AC-containing bait palatability was low, and the cats were very reluctant to eat it, even when it contained low concentrations of AC.^17^ Cornwell reported that cats did not eat AC bait, even when fasted for 36 h.^8^ This could indicate that the cats in the present study were secondarily poisoned, rather than having eaten bait directly. However, hazard analysis of AC deemed the risk for secondary poisoning to mammal predators as ‘negligible’.^18^ In summary, with the available data, it was not possible to know how the cats were exposed to AC.

Owing to its retrospective nature there are weaknesses in this study. Samples were obtained opportunistically, and blood sampling was performed at different points in time in relation to the progression of intoxication. This limitation likely influenced the measured concentrations in each sample. Our data show a clear correlation between clinical severity and AC concentration, which is expected with a dose-dependent toxin. Some individual differences between cats’ ability to metabolise and excrete the toxin may however, influence the clinical severity in relation to AC concentration. Although all signs of intoxication were registered, including those reliably reported from the caretaker, the study was dependent on the extent of the history-taking by the attending clinicians upon admission, as well as during stationary care. As the study population consisted of outdoor cats, some clinical signs may also have gone unobserved by the caregiver. Furthermore, the confirmed cases in this study were all sampled in 2019 and 2020, when the clinical records generally included more detailed descriptions about which signs were present or absent due to increased awareness. Earlier records (2014–2018) are often less elaborate, and some signs may therefore be under-reported in the unconfirmed cases. As a direct, quantitative method of AC detection was not used in the study by Segev et al,^12^ a comparison between differences in blood concentrations of AC – and its impact on the prevalence of clinical signs and intoxication severity – was not possible.

In the present study, mortality and duration of hospitalisation was not dependent on the severity of clinical signs, indicating that intoxication severity is not indicative of prognosis. Our data suggest that cats with AC poisoning will rarely succumb when given proper supportive care in a timely manner.

## Conclusions

This study provides a clinical description of AC intoxication in cats, describing 25 confirmed and 78 suspected cases of AC poisoning. Cats with AC intoxication have an excellent prognosis for complete recovery, providing that supportive care is given in a timely manner. When admitting an outdoor cat with acute onset of described neurological signs, no history of trauma and possible AC exposure, intoxication should be included in the list of differential diagnoses. This may influence both clinical decision-making, diagnostic work-up and client communication. A severe clinical picture upon presentation could be misinterpreted as a grave prognosis and both clinical and client awareness about AC poisoning may avoid unnecessary euthanasia.

## Supplemental Material

Supplemental Material Click here for additional data file. Definitions of clinical signs.
