# Supplementary material for: Alpha-chloralose poisoning in cats: clinical findings in 25 confirmed and 78 suspected cases
Source: J Feline Med Surg. 2022 Jun 27;24(10):e324–9. doi: 10.1177/1098612X221107787 (PMC9510939; doi:10.1177/1098612X221107787)
Supplement: Supplemental Material [file sj-docx-1-jfm-10.1177_1098612X221107787.docx]

Clinical definitions

**Coma** - non-ambulatory, obtunded, unconscious and unresponsive including to nociceptive stimuli.

**Stupor** - non-ambulatory, obtunded, unresponsive to external stimuli except nociceptive stimuli. May have some degree of consciousness.

**Somnolence** - impaired reactions to external stimuli and reduced awareness.

**Seizures** - tonic/clonic or tonic epileptiform seizures in unconscious, non-ambulatory patient.

**Cranial nerve affection** - including miosis, mydriasis, anisocoria, impairment of pupil light reflex, dazzle or menace, facial nerve paralysis, vestibular syndromes, ptyalism, Horner’s syndrome etc.

**Vision impairment** - signs indicating reduced/impaired ability to process visual stimuli. These patients are also noted as cranial nerve affection.

**Ataxia** - decreased ability to coordinate movements, including proprioceptive, cerebellar and vestibular ataxia.

**Hypotension** - average systolic pressure ≤100 mmHg or mean arterial pressure ≤80 mmHg.

**Hypothermia** - body temperature ≤37^o^C.

**Bradycardia** - heart rate ≤140/min.

**Bradypnea** - respiratory rate ≤15/min.

**Other respiratory alteration** - choppy breathing pattern, intermittent apnoea or other abnormal breathing patterns.

**Hyperesthesia** - exaggerated reactions to external stimuli such as sound, light or touch.

**Tremor** - rhythmic oscillating movement generalised or localised, especially involving ears, whiskers and muzzle.

**Behavioural changes** - in patients with normal consciousness. Includes signs of hallucinations, extreme polyphagia, desorientation, euphoria, dysphoria, hypervigilance, compulsive behaviour or aggression in a patient who is normally calm/timid
